# Supplementary material for: A Mediterranean Alexandrium taylorii (Dinophyceae) Strain Produces Goniodomin A and Lytic Compounds but Not Paralytic Shellfish Toxins
Source: Toxins (Basel). 2020 Sep 1;12(9):564. doi: 10.3390/toxins12090564 (PMC7551950; doi:10.3390/toxins12090564)
Supplement: Supplementary file 1 [file toxins-12-00564-s001.pdf]

Supplementary Materials: A Mediterranean *Alexandrium taylorii* (Dinophyceae) Strain Produces Goniodomin A and Lytic Compounds but Not Paralytic Shellfish Toxins

Urban Tillmann, Bernd Krock, Stephan Wietkamp, and Alfred Beran

Table S1. Reference LSU and ITS DNA sequences for *A. taylorii*.

Strain	Geographic origin	Reference	Gene	Genbank Accession no.
AY10T	Adriatic Sea, Lagoon of Marano, Italy	Penna et al. [1]	ITS	AM296013.1
AY2T	Adriatic Sea, Lagoon of Marano, Italy	John et al. [2]	LSU	AJ535348
AY4T	Adriatic Sea, Lagoon of Marano, Italy	John et al. [2]	LSU	AJ535349
AY7T	Adriatic Sea, Lagoon of Marano, Italy	Penna et al. [1]	ITS	AM296012.1
AY1T	Adriatic Sea, Lagoon of Marano, Italy	John et al. [2], Penna et al. [1]	LSU<br>ITS	AJ535347<br>AM296011.1
CBA-1	Aegean Sea, Kavala, Greece	Penna et al. (unpublished)	ITS	AJ416856.1
AT4	Ionian Sea, Siracusa, Italy	Penna [3]	ITS	AJ251653.1
B2	Tyrrhenian Sea, Vulcano, Italy	Penna et al. (unpublished)	ITS	AJ300451.1
AV-8	Catalan Sea, La Fosca, Spain	Penna [3]	ITS	AJ251654.1
Field sample	Adriatic Sea, Cesenatico, Italy	Penna et al. (unpublished)	ITS	AM296010.1
Temporary-cyst	Tyrrhenian Sea, Vulcano, Italy	Penna et al. (unpublished)	ITS	AJ291785
VGOE6	Catalan Sea, La Fosca, Spain	Penna et al. [1]	ITS	AM236856
VGO705	Catalan Sea, Paguera, Spain	Penna et al. [1]	ITS	AM296014.1
Atay99Shio-01	Shioya Bay, Okinawa Pref., Japan	Nagai and Itakura [4]	LSU	AB607263.1
Atay99Shio-02	Shioya Bay, Okinawa Pref., Japan	Nagai and Itakura [4]	LSU	AB607264.1
Atay99Shio-03	Shioya Bay, Okinawa Pref., Japan	Nagai and Itakura [4]	LSU	AB607265.1
Atay99Shio-06	Shioya Bay, Okinawa Pref., Japan	Nagai [5]	ITS[#]	AB841262.1
AY7T	Adriatic Sea, Lagoon of Marano, Italy	**This study**	LSU<br>ITS	MT643180<br>MT644478

[#]note that in GenBank this entry has the header "*Alexandrium taylorii* genes for 18S rRNA, ITS1, 5.8S rRNA, ITS2, 28S rRNA, partial and complete sequence, strain: Atay99Shio-06" but in fact contains only 538 bp ITS sequence information.

References

1. Penna, A.; Fraga, S.; Maso, M.; Giacobbe, M.G.; Bravo, I.; Garcés, E.; Vila, M.; Bertozzini, E.; Andreoni, F.; Luglie, A.; et al. Phylogenetic relationships among the Mediterranean *Alexandrium* (Dinophyceae) species based on sequences of 5.8S gene and Internal Transcript Spacers of the rRNA operon. *Eur. J. Phycol.* **2008**, *43*, 163–178.

2. John, U.; Fensome, R.A.; Medlin, L.K. The application of a molecular clock based on molecular sequences and the fossil record to explain biogeographic distributions within the *Alexandrium tamarense* "species complex" (Dinophyceae). *Mol. Biol. Evol.* **2003**, *20*, 1015–1027.
3. Penna, A. Blooms of *Alexandrium taylori* (Dinophyceae) in the Mediterranean: A preliminary molecular analysis of different isolates. In *Harmful Algal Blooms 2000, Proceedings of the 9TH International Conference on hatrmful Algal Blooms, Hobart, Australia*; Hallegraeff, G.M., Blackburn, S.I., Bolch, C.J.S., Lewis, R.J., Eds.; Unesco: Paris, France, 2001; pp. 218–221.
4. Nagai, S.; Itakura, S. Specific detection of the toxic dinoflagellates *Alexandrium tamarense* and *Alexandrium catenella* from single vegetative cells by a loop-mediated isothermal amplification method. *Mar. Genom.* **2012**, *7*, 43–49.
5. Nagai, S. Species-specific detection of six *Alexandrium* species from single vegetative cells by a loop-mediated isothermal amplification method. *DNA Test.* **2013**, *5*, 33–46.

Table S2. MS parameters of PST and GC toxin analysis.

Parameter	ESI+	ESI-
Soft Transmission Mode	enabled	disabled
Capillary (kV)	0.5	2.5
Cone (V)	10	10
Source Offset (V)	30	30
Source Temperature (°C)	150	150
Desolvation Temperature (°C)	600	600
Cone Gas Flow (L h^{-1})	150	150
Desolvation Gas Flow (L h^{-1})	1000	1000
Collision Gas Flow (mL min^{-1})	0.15	0.15
Nebuliser Gas (bar)	7	7

Table S3. MS/MS parameters of lipophilic toxin analysis.

Parameter	ESI+
Capillary (kV)	3.0
Cone (V)	40
Source Offset (V)	20
Source Temperature (°C)	150
Desolvation Temperature (°C)	600
Cone Gas Flow (L h^{-1})	150
Desolvation Gas Flow (L h^{-1})	1000
Collision Gas Flow (mL min^{-1})	0.15
Nebuliser Gas (bar)	7
Collision energy (eV)	40

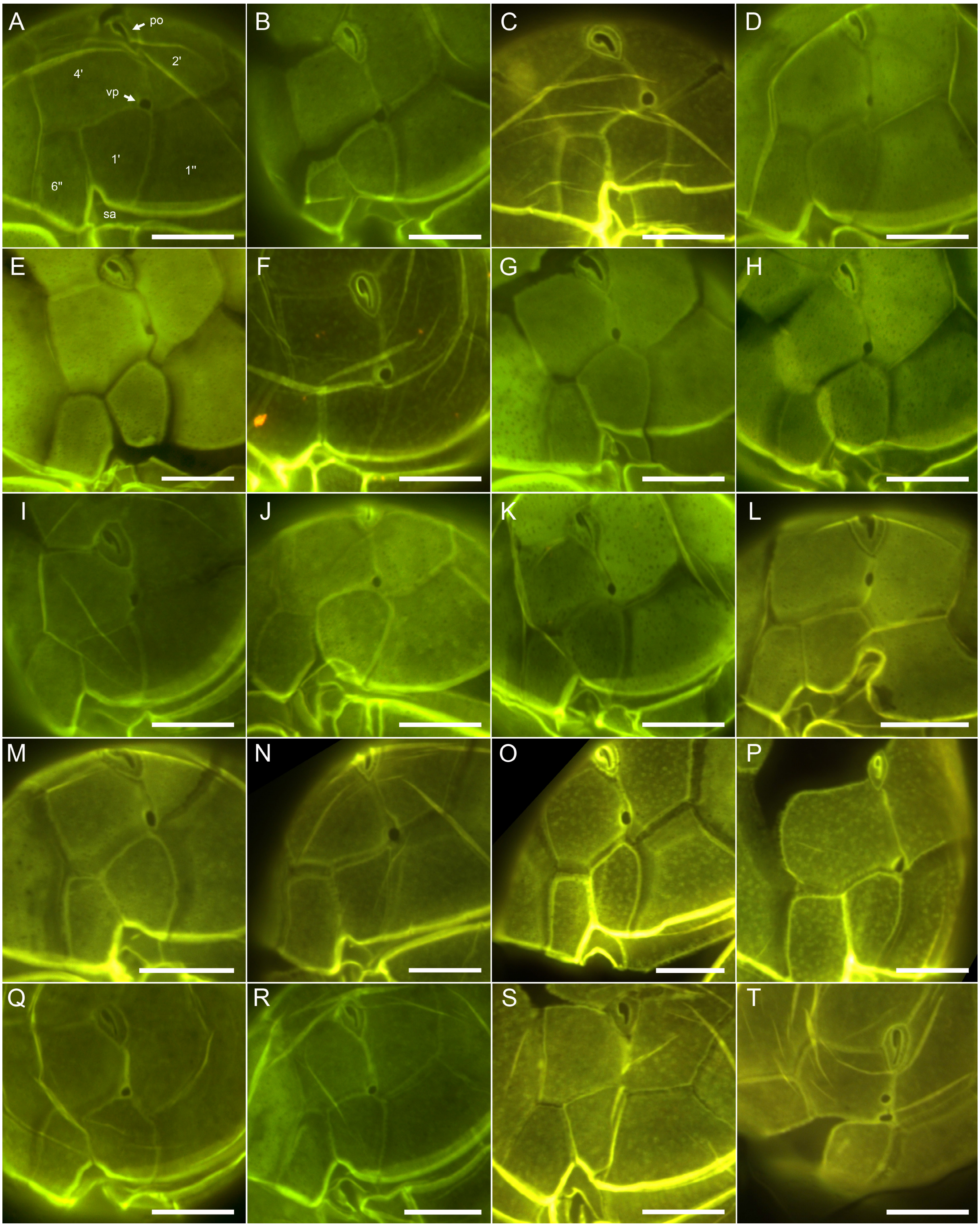

Figure S1. *Alexandrium taylorii* AY7T. Detailed ventral views of epithecal plates of different Lugol-fixed cells stained with Solophenyl Flavine and viewed with epifluorescence and blue light excitation to illustrate shape variability of plates 1' and 6'' and variability in position of the ventral pore (vp). Note that in (S) no vp could be identified, whereas in (T) two vp were present. Plate label exemplarily shown in (A). Scale bars = 10 μm.

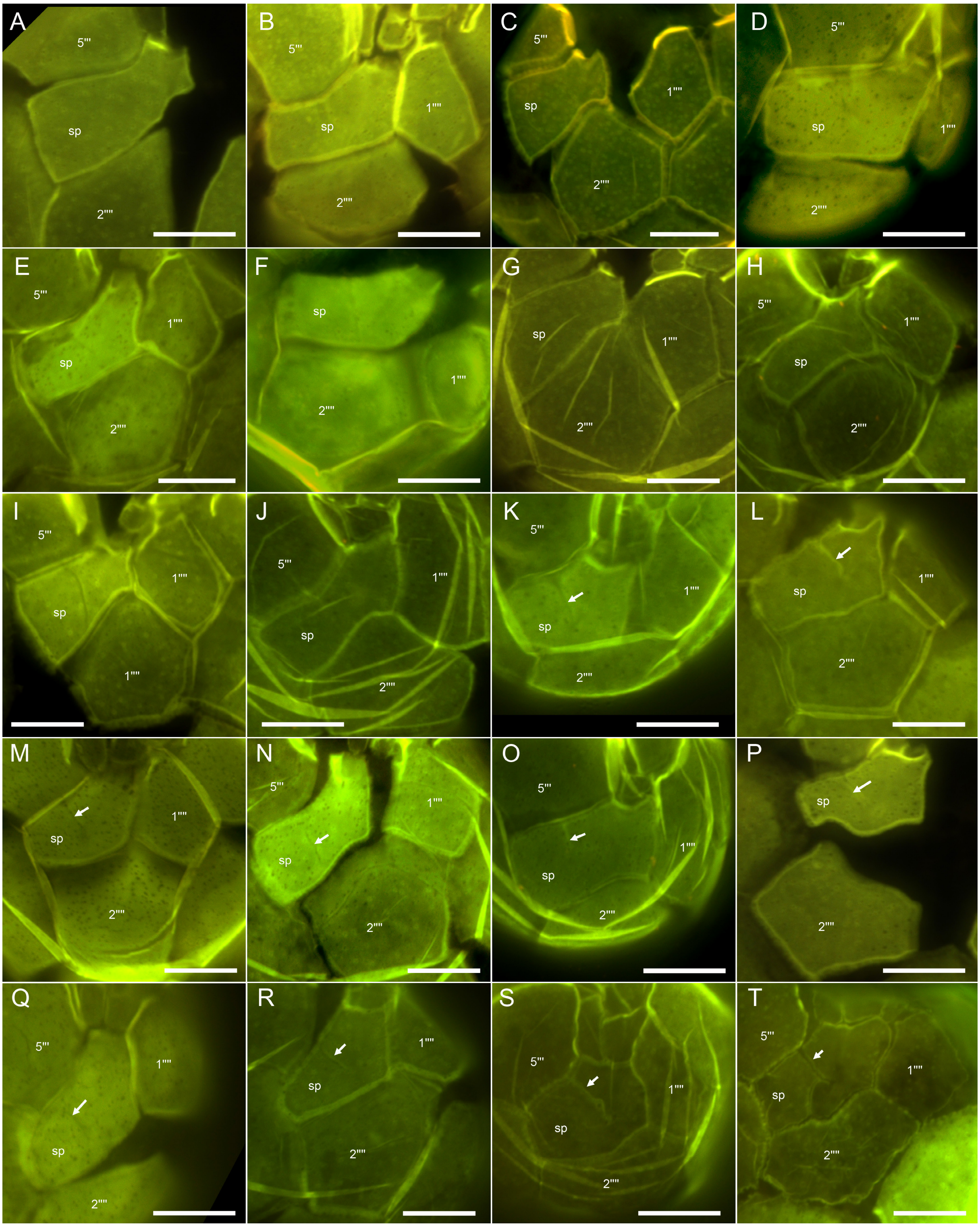

Figure S2. *Alexandrium taylorii* AY7T. Detailed ventral or antapical views of hypothecal plates of different Lugol-fixed cells stained with Solophenyl Flavine and viewed with epifluorescence and blue light excitation to illustrate shape variability of the posterior sulcal plate sp. Note the faint groove that extend from the right margin of sp in K–T (arrows) which occasionally ended in a small pore (S) or continued to the left margin (T). Scale bars = 10 µm.

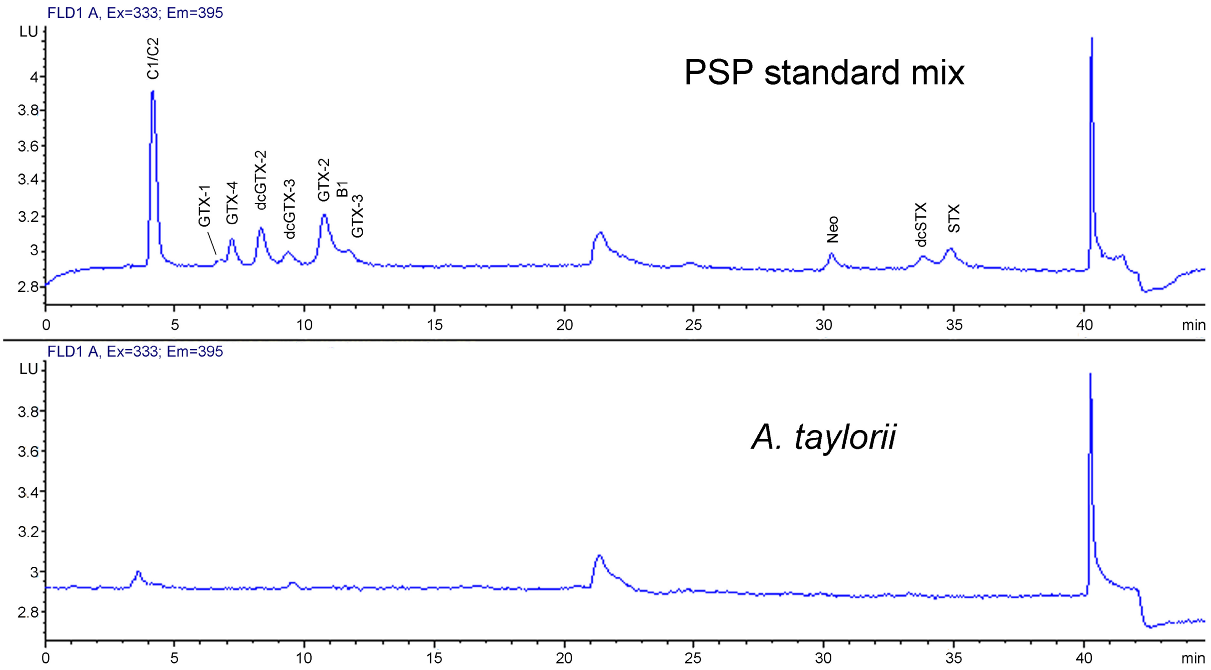

Figure S3. LC-FLD chromatograms of a PST standard mix (upper panel) and the *Alexandrium taylorii* extract (lower panel). Concentrations of the PST standard solution are the following: C1: 100.3 pg μL^{-1}; C2: 28.6 pg μL^{-1}; GTX1: 205.4 pg μL^{-1}; GTX4: 54.7 pg μL^{-1}; dcGTX2: 16.1 pg μL^{-1}; dcGTX3: 4.5 pg μL^{-1}; GTX2: 16.3 pg μL^{-1}; GTX3: 5.4 pg μL^{-1}; B1: 26.3 pg μL^{-1}; NEO: 100 pg μL^{-1}; dcSTX: 12.8 pg μL^{-1}; STX: 14.7 pg μL^{-1}.

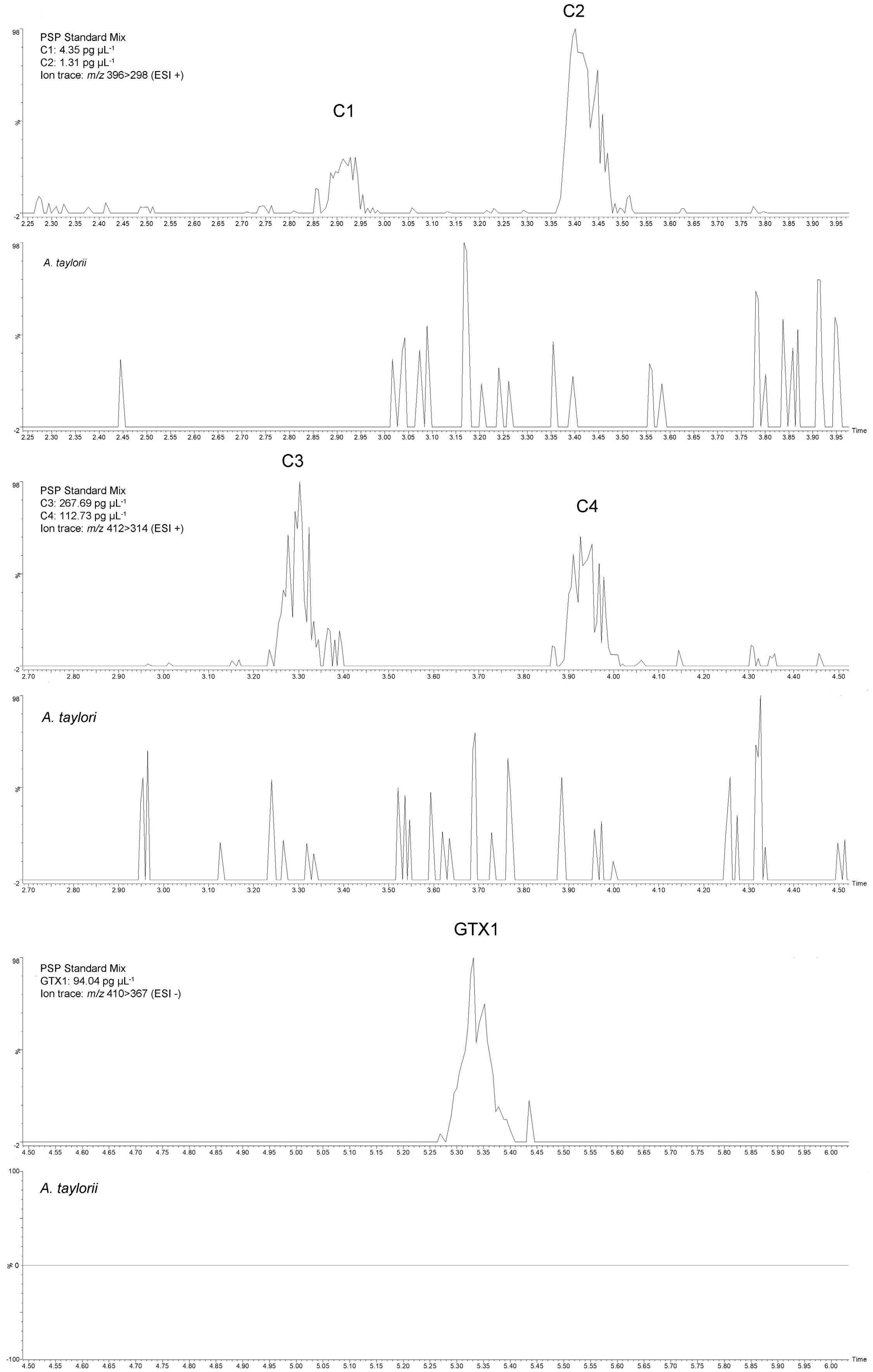

Figure S4. Extracted Ion chromatograms of a PST standard mix (upper panels) and the *A. taylorii* extract (lower panels).

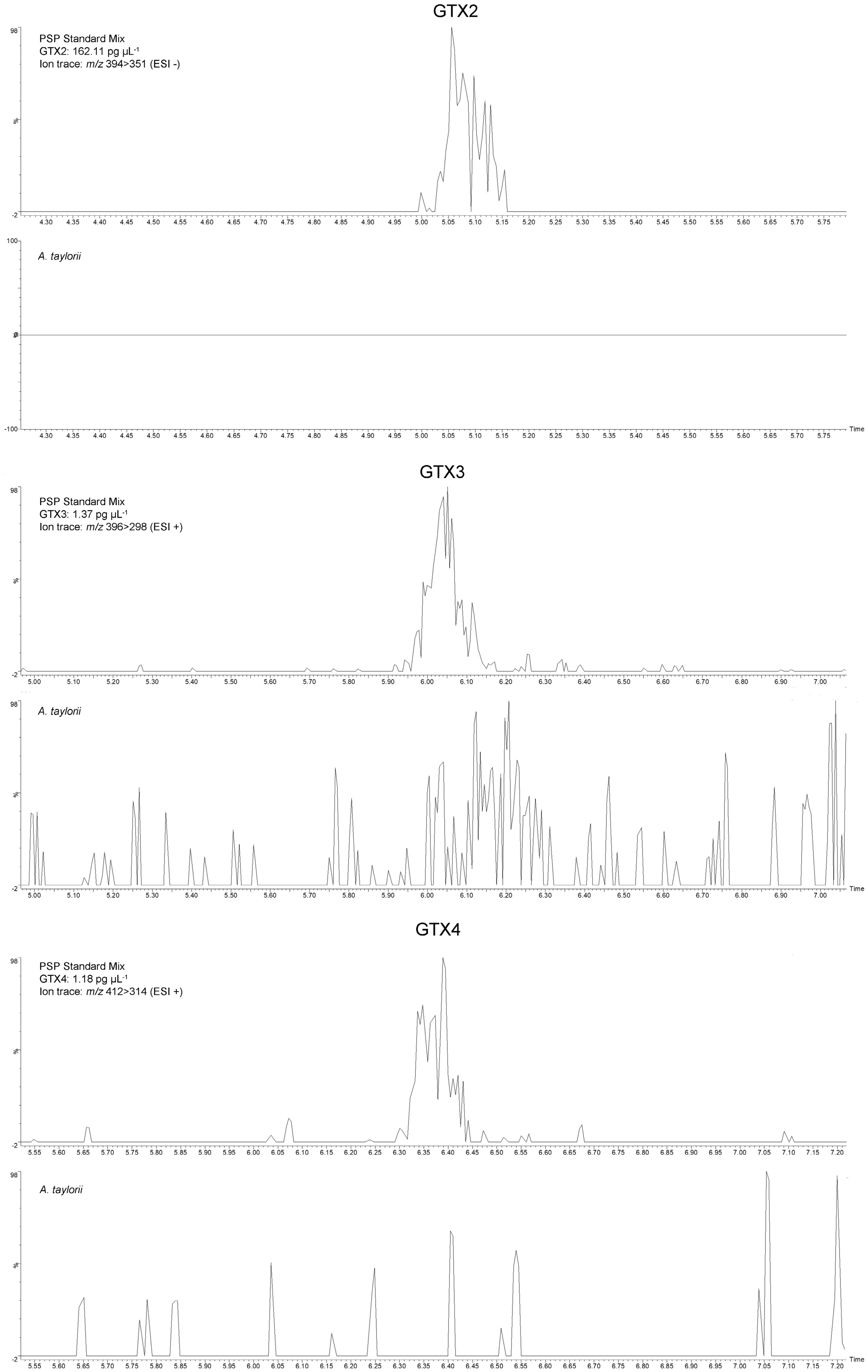

Figure S4. continued.

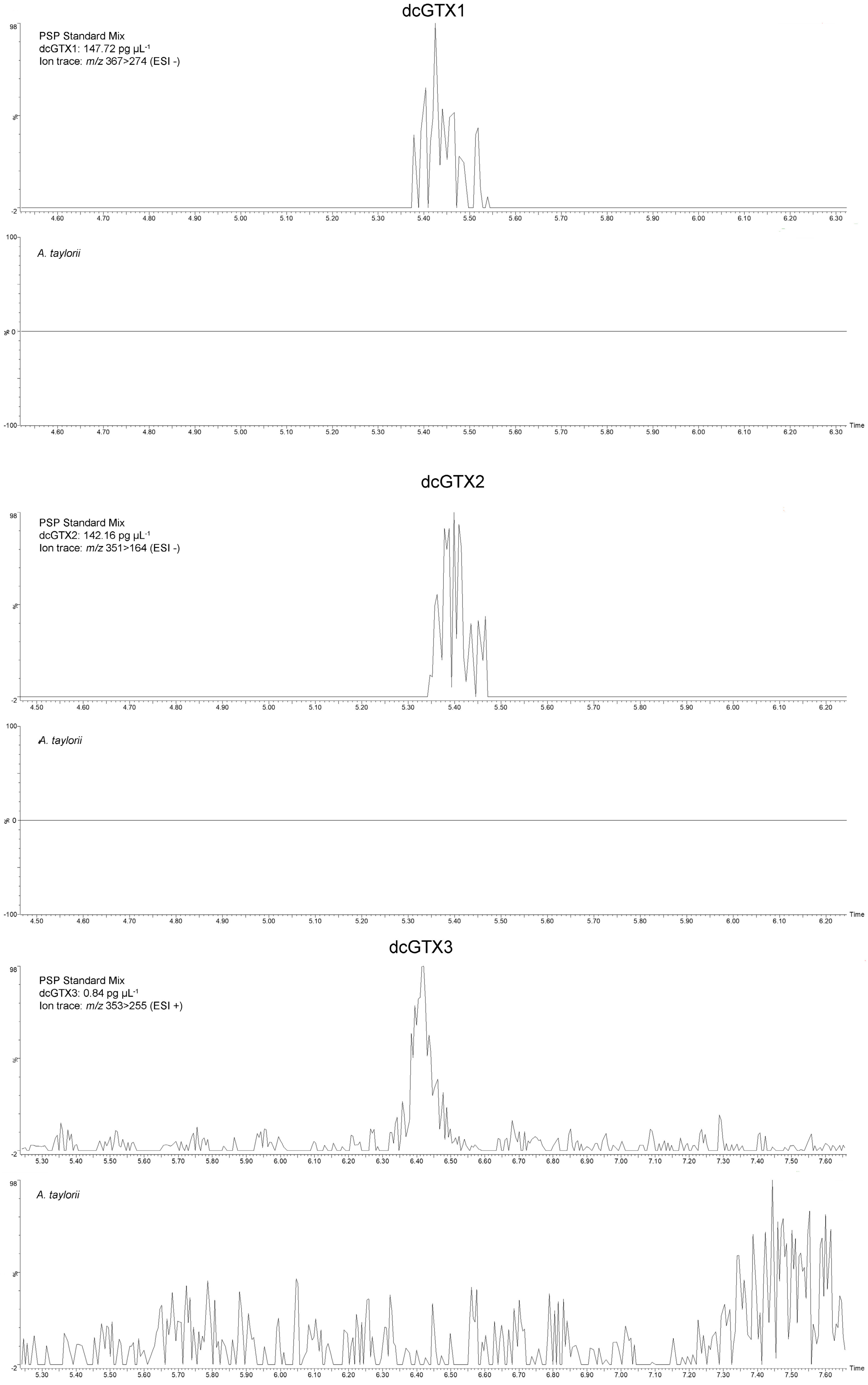

Figure S4. continued.

dcGTX4

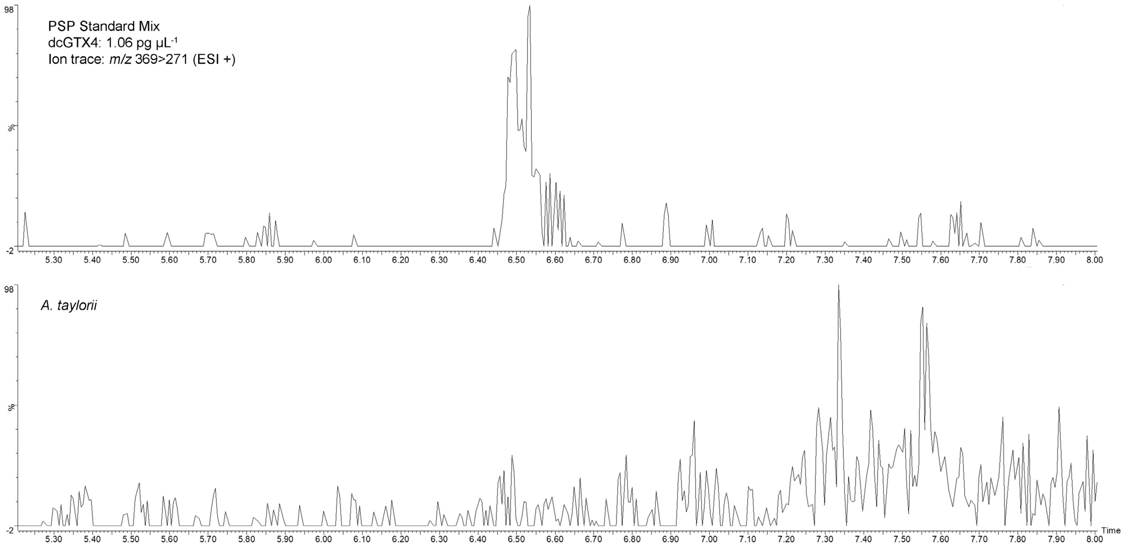

B1

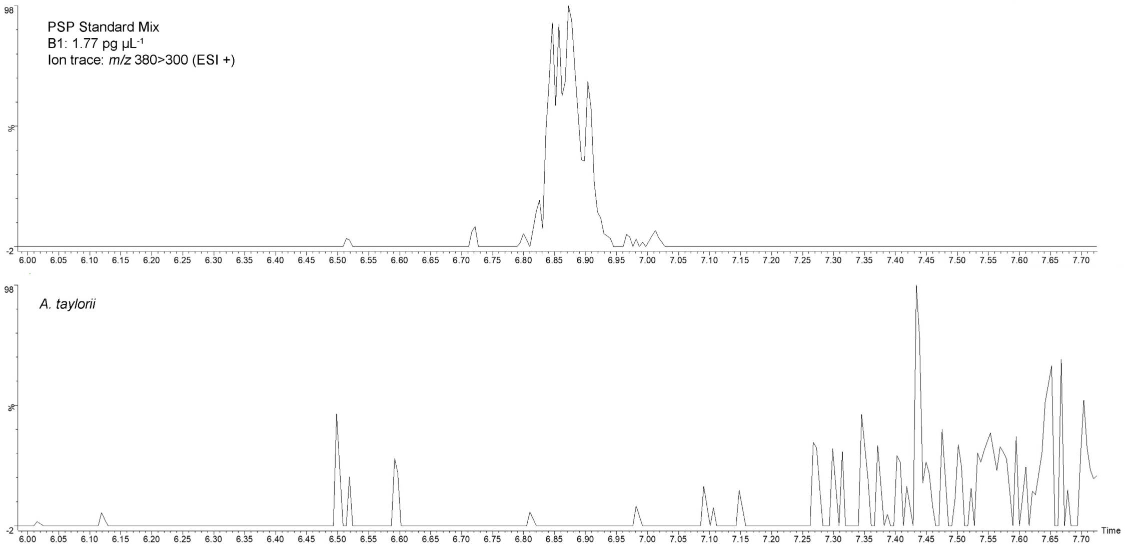

B2

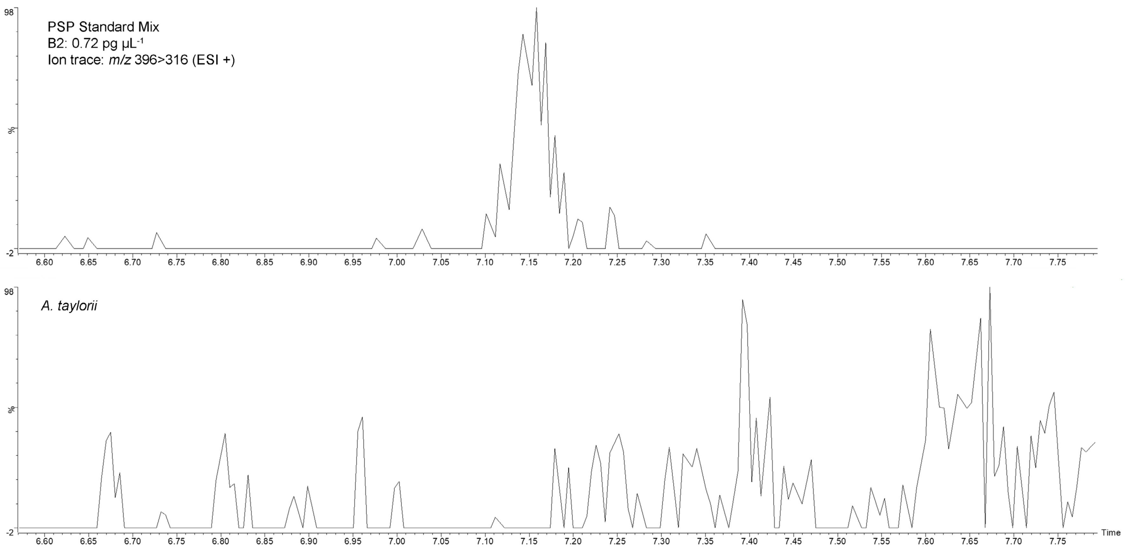

Figure S4. continued.

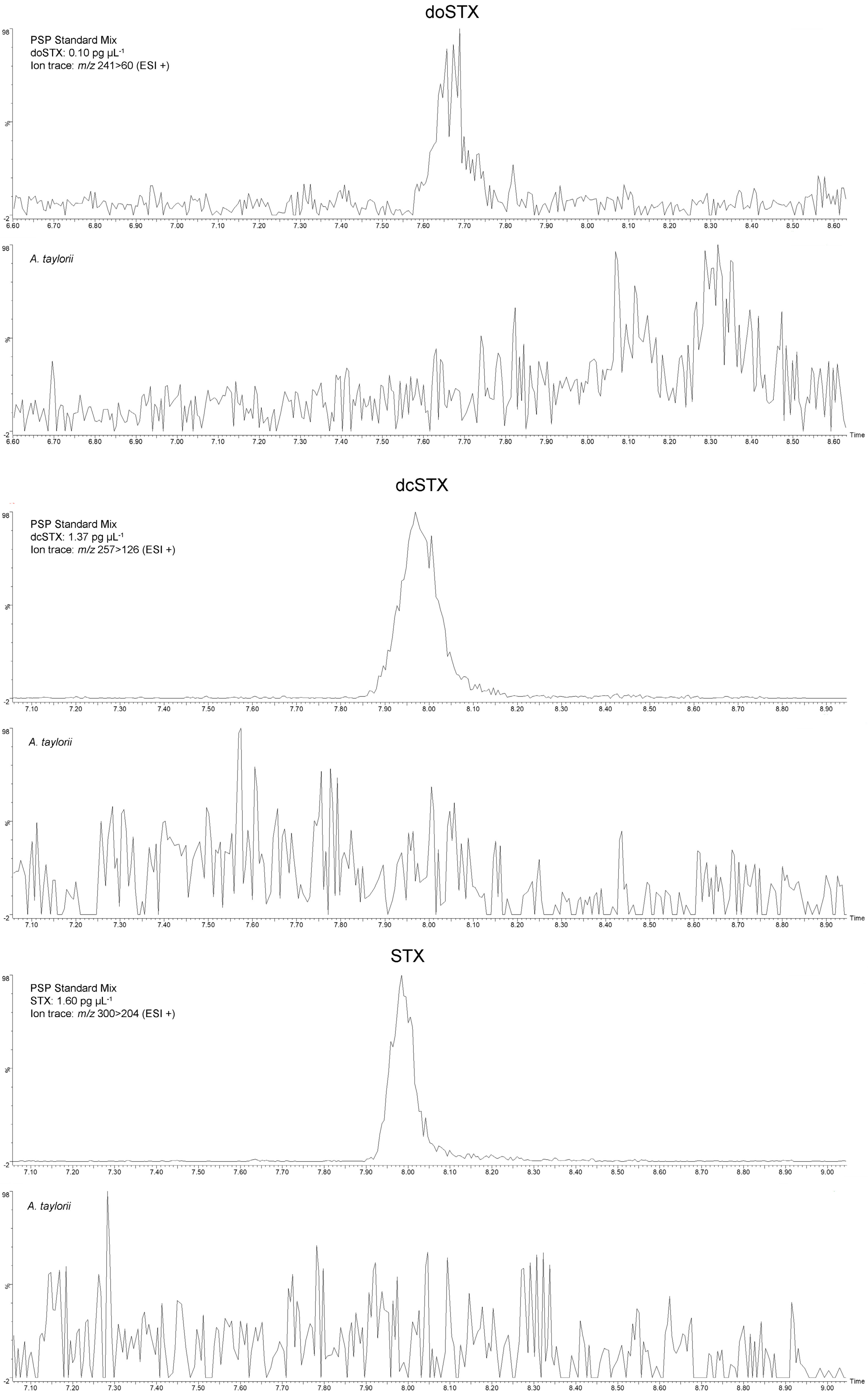

Figure S4. continued.

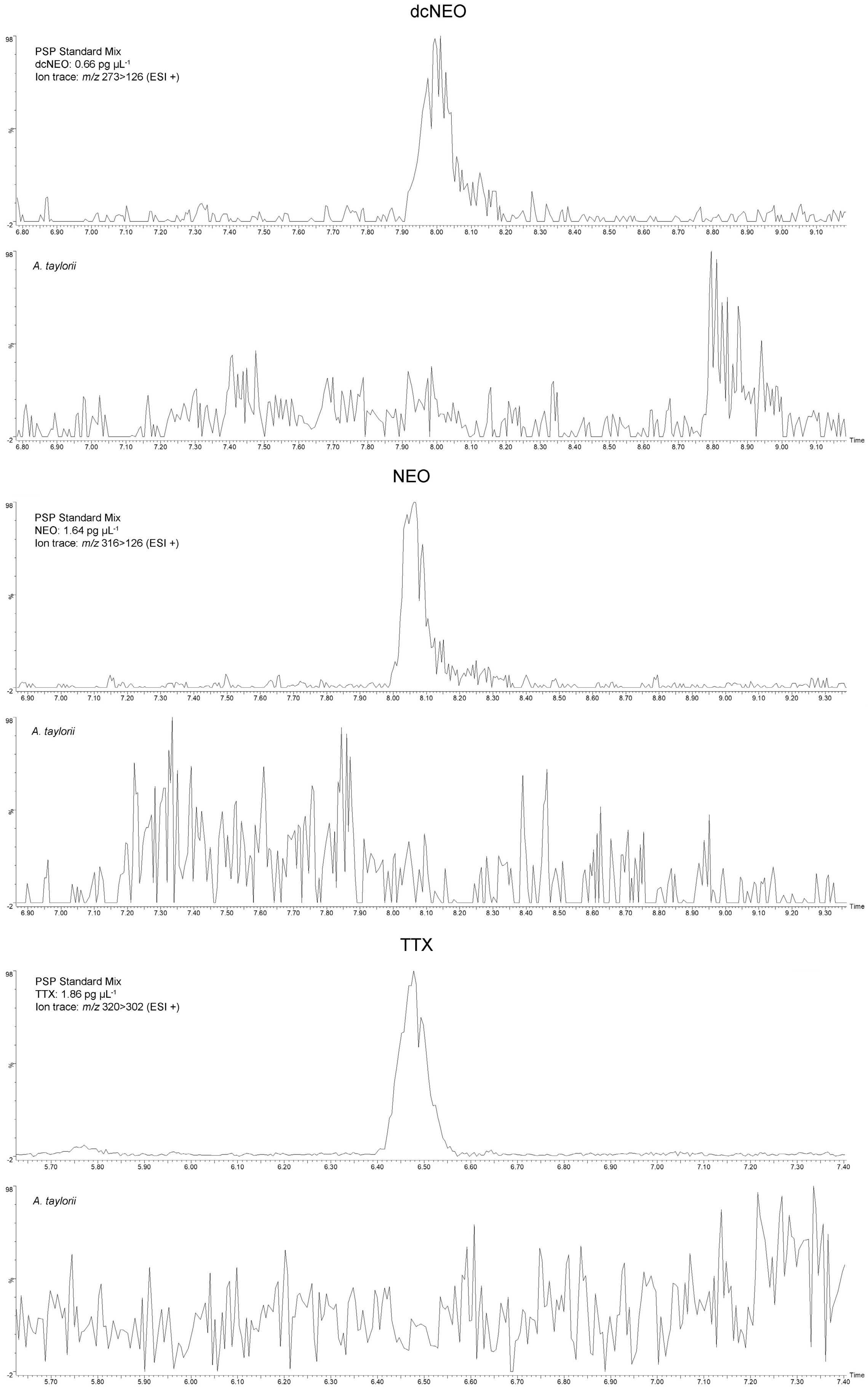

Figure S4. continued.
